# Platelet Activation and Thrombus Formation over IgG Immune Complexes Requires Integrin αIIbβ3 and Lyn Kinase

**DOI:** 10.1371/journal.pone.0135738

**Published:** 2015-08-20

**Authors:** Huiying Zhi, Jing Dai, Junling Liu, Jieqing Zhu, Debra K. Newman, Cunji Gao, Peter J. Newman

**Affiliations:** 1 Blood Research Institute, BloodCenter of Wisconsin, Milwaukee, Wisconsin, United States of America; 2 Ruijing Hospital Affiliated Shanghai Jiao Tong University School of Medicine, Shanghai, People’s Republic of China; 3 Department of Biochemistry and Molecular Cell Biology, Shanghai Key Laboratory of Tumor Microenvironment and Inflammation, Shanghai JiaoTong University School of Medicine, Shanghai, China; 4 Department of Biochemistry, Medical College of Wisconsin, Milwaukee, Wisconsin, United States of America; 5 Department of Microbiology, Medical College of Wisconsin, Milwaukee, Wisconsin, United States of America; 6 Department of Pharmacology and Toxicology, Medical College of Wisconsin, Milwaukee, Wisconsin, United States of America; 7 Chronic Disease Research Institute, Department of Nutrition and Food Hygiene, Zhejiang University School of Public Health, Hangzhou, China; 8 Department of Cell biology, Medical College of Wisconsin, Milwaukee, Wisconsin, United States of America; University of Leuven, BELGIUM

## Abstract

IgG immune complexes contribute to the etiology and pathogenesis of numerous autoimmune disorders, including heparin-induced thrombocytopenia, systemic lupus erythematosus, rheumatoid- and collagen-induced arthritis, and chronic glomerulonephritis. Patients suffering from immune complex-related disorders are known to be susceptible to platelet-mediated thrombotic events. Though the role of the Fc receptor, FcγRIIa, in initiating platelet activation is well understood, the role of the major platelet adhesion receptor, integrin αIIbβ3, in amplifying platelet activation and mediating adhesion and aggregation downstream of encountering IgG immune complexes is poorly understood. The goal of this investigation was to gain a better understanding of the relative roles of these two receptor systems in immune complex-mediated thrombotic complications. Human platelets, and mouse platelets genetically engineered to differentially express FcγRIIa and αIIbβ3, were allowed to interact with IgG-coated surfaces under both static and flow conditions, and their ability to spread and form thrombi evaluated in the presence and absence of clinically-used fibrinogen receptor antagonists. Although binding of IgG immune complexes to FcγRIIa was sufficient for platelet adhesion and initial signal transduction events, platelet spreading and thrombus formation over IgG-coated surfaces showed an absolute requirement for αIIbβ3 and its ligands. Tyrosine kinases Lyn and Syk were found to play key roles in IgG-induced platelet activation events. Taken together, our data suggest a complex functional interplay between FcγRIIa, Lyn, and αIIbβ3 in immune complex-induced platelet activation. Future studies may be warranted to determine whether patients suffering from immune complex disorders might benefit from treatment with anti-αIIbβ3-directed therapeutics.

## Introduction

IgG immune complexes contribute to the etiology and pathogenesis of a number of autoimmune disorders, including heparin-induced thrombocytopenia [1], systemic lupus erythematosus [2,3], and collagen-induced/rheumatoid arthritis [4]. Patients with immune complex-related disorders are known to be hypercoagulable [5], and susceptible to both thrombocytopenia [6,7] and thrombosis [8,9]. These disorders are thought to be precipitated, at least in part, by platelets that have become activated via their interaction with autoimmune antibody/antigen complexes—an event that was shown almost 50 years ago to induce secretion of platelet granule constituents [10], and that is now known to be mediated by the binding of the Fc region of IgG-containing immune complexes to the platelet cell surface Fc receptor, FcγRIIa.

FcγRIIa is a member of the immunoglobulin gene superfamily comprised of an extracellular domain that binds the Fc region of IgG, a single pass transmembrane domain, and a cytoplasmic tail that contains two YxxL immune receptor tyrosine-based activation motifs (ITAMs) [11,12]. While FcγRIIa exhibits only low-affinity for monomeric IgG, it binds with high affinity to the Fc region of antigen-bound IgG immune complexes [11,13]. FcγRIIa is the only Fc receptor on human platelets, and is not expressed in mice [14]. Its cross-linking results in activation of associated Src-family kinases that phosphorylate the ITAM tyrosines, which act as a docking site for the SH2 domain-containing tyrosine kinase, Syk [15]. Activation of Syk, in turn, promotes an intracellular signaling cascade that eventually leads to phosphorylation and activation of phospholipase C (PLC) γ2 [16], resulting in calcium mobilization, granule secretion, integrin activation, platelet aggregation, and thrombus formation.

In addition to its role as a receptor for IgG-containing immune complexes, FcγRIIa appears to be capable of promoting a number of other functions in platelets, most notably as an amplifier of integrin αIIbβ3-mediated platelet activation [17,18], and in cooperating with this integrin to mediate platelet activation by tumor cells [19] and certain strains of bacteria [20]. Interestingly, although FcγRIIa was found to mediate the initial attachment of FcγRIIa-transfected HEK293 to immobilized immune complexes, sustained signaling downstream of attachment required co-expression of the integrin α_M_β_2_ (Mac-1) [21]. Thus, at least in transfected cell lines, the ability of FcγRIIa to send productive activation signals into a cell requires integrin signaling as well. The purpose of the present investigation was to determine whether there is functional coupling between FcγRIIa and αIIbβ3 when platelets encounter immobilized IgG. Our results help define the molecular requirements for platelet activation and thrombus formation in patients suffering from IgG immune complex disorders, and have potential therapeutic implications for treating and/or preventing the thrombotic complications associated with immune complex disorders.

## Materials and Methods

### Reagents and antibodies

The hybridoma producing the anti-FcγRIIa mAb, IV.3, was obtained from the American Type Culture Collection (Manassas, VA). Antibodies specific for Syk, Src and β-actin, and bovine serum albumin were purchased from Santa Cruz Biotechnology. Antibodies against focal adhesion kinase were from Thermo Scientific. Antibodies specific for Syk (phosphorylated tyrosine 525/526), Src (phosphorylated tyrosine 416) and Fak (phosphorylated tyrosine 397) were from Cell Signaling Technology. Anti-phosphotyrosine mAb 4G10 was purchased from Millipore. Fab fragments were prepared using a kit from Pierce Biotechnology. Phosphatase inhibitor cocktail was purchased from EMD Chemicals. Halt Protease inhibitor cocktail was purchased from Thermo Scientific. Human IgG was from Jackson ImmunoResearch Laboratories. Human fibrinogen was from Enzyme Research Laboratories Inc. Lyn and Fyn were obtained from Life Technologies. Src was purchased from Enzo Life Sciences.

### Mice

Mice were maintained in the Biological Resource Center at the Medical College of Wisconsin (MCW). All animal protocols were approved by the MCW Institutional Animal Care and Use Committee. FcγRIIa transgenic mice [14] were littermates on a C57BL/6J background. Lyn^-/-^ mice (C57BL/6 background) were from the Jackson Laboratories, β3^-/-^ mice (C57BL/6 background) were a gift of David A. Wilcox (MCW). Fyn^-/-^ mice (129 background) were from Roy L. Silverstein (MCW). FcγRIIa mice were bred with Lyn-deficient mice (Lyn^-/-^), Fyn-deficient mice (Fyn^-/-^) and β3 deficient mice (β3^-/-^) to obtain double-heterozygous mice that were used to establish Lyn^-/-^/FcγRIIa^pos^, Lyn^+/+^/FcγRIIa^pos^, Fyn^-/-^/FcγRIIa^pos^, Fyn^+/+^/FcγRIIa^pos^, β3^-/-^/FcγRIIa^pos^, β3^+/+^/FcγRIIa^pos^ colonies. Mouse genotypes were verified by PCR amplification of genomic DNA. Expression of FcγRIIa, and lack of Lyn, Fyn, and β3 were confirmed by Western blot analysis of platelet lysates. Platelet counts of the Lyn^-/-^/FcγRIIa^pos^, Fyn^-/-^/FcγRIIa^pos^ mice were similar to that of wild-type controls.

### Blood collection

Ethical approval was obtained from the institutional review board of the BloodCenter of Wisconsin in accordance with the Declaration of Helsinki. All study members provided informed, written consent to participate. Blood samples from a Type I Glanzmann thrombasthenic (GT) patient carrying an L924A point mutation in integrin αIIb, and from healthy volunteers free from medication for two weeks was collected into 90 mM PPACK (D-phenylalanyl-L-prolyl-L-arginine chloromethyl ketone). Mouse blood was drawn from the inferior vena cava of anesthetized mice into 3.8% sodium citrate (1/10 volume) or PPACK/heparin.

### Platelet spreading assays

Platelets were obtained from 3.8% sodium citrate anticoagulated whole blood and added to 8-chamber glass tissue-culture slides (Becton Dickinson) that had been coated with human IgG (25 μg/ml) or human fibrinogen (25 μg/mL). Fibrinogen and BSA were precleared using protein G Sepharose (GE Healthcare Bio-Sciences) to remove any traces of contaminating IgG. Spreading assays were performed as previously described [18]. Briefly, 200 μl of washed platelets at a concentration of 2.5x10^7^/mL were allowed to adhere to either immobilized fibrinogen or IgG for 30 minutes. Adherent platelets were fixed with 2% paraformaldehyde, permeabilized with 0.1% Triton X-100, and stained with phalloidin tetramethylrhodamine isothiocyanate. Images analyzed using Metamorph software (Universal Imaging). Statistical analysis of the area occupied by spread platelets was performed using a 2-tailed Student t-test for unpaired samples. For biochemical analysis, platelets were incubated at 37°C for 30 minutes on 10 cm tissue-culture dishes and lysed with 30 mM HEPES [pH 7.4], 300 mM NaCl, 20 mM EGTA, 0.2 mM MgCl_2_, 2% Triton X-100) containing protease and phosphatase inhibitor cocktails, and subjected to immunoblot analysis.

### In vitro thrombus formation under flow conditions

Thrombus formation was evaluated by a whole-blood perfusion assay over immobilized human IgG under venous shear conditions as previously described [18]. Briefly, heparin/PPACK-anticoagulated whole blood labeled with mepacrine (CalBiochem) was perfused over IgG-coated Vena8 Fluoro+ Biochip microchannels (Cellix Ltd) in the presence or absence of αIIbβ3 antagonists. Epifluorescence microscopic images of platelet adhesion and thrombus formation were acquired by in real time at one frame per second. Thrombus formation was determined as the mean percentage of total area covered by thrombi and as the mean integrated fluorescence intensity per μm^2^. Image analysis was performed using Metamorph.

### PF4 release assay

Washed platelets from αIIbβ3^-/-^/FcγRIIa^pos^ and αIIbβ3^+/+^/FcγRIIa^pos^ mice were allowed to spread on glass slides that had been coated with BSA, 25 μg/ml fibrinogen, or 25 μg/mL IgG for 30 minutes. After spreading, the platelets were removed by centrifugation and the secreted PF4 quantified using a Quantikine ELISA kit (R&D systems).

### CHO cell transfection and spreading assay

CHO-K1 cells stably expressing full-length human αIIb and β3 [22] were additionally transfected with pCMV FcγRIIa IRES neo (Addgene) using Lipofectamine LTX and PLUS Reagent. The expression levels of αIIb, β3, and the FcγRIIa were confirmed by Western-blot. Spreading assays were performed by adding cells to glass slides that had been coated with either 25 μg/ml IgG or fibrinogen in the presence or absence of 250 μg/ml soluble fibrinogen. Phase contrast images of CHO cells were taken 45 minutes later.

### Mass spectrometric analysis of phosphorylated FcγRIIa cytoplasmic domain constructs

The region encoding amino acid residues 206–282 of the FcγRIIa cytoplasmic domain was PCR-amplified from pCMV FcγRIIa IRES neo and cloned into the bacterial expression vector pQE30-GB1 (Qiagen) in front of a histidine tag. The resulting construct was transduced into E. coli BL21 cells, induced with IPTG and purified from bacterial lysates using a nickel-Sepharose column (GE Healthcare Life Sciences). For kinase assays, recombinant FcγRIIa cytoplasmic domain proteins (1 mM) were incubated with Src, Lyn or Fyn in kinase assay buffer (250 μM ATP, 1 mM EGTA, 10 mM MgCl_2_, 0.01% Brij 35, and 250 μM Na_3_VO_4_) for 60 min at 30°C and then boiled in the presence of an equal volume of 2× SDS-PAGE sample reducing buffer. The resulting products were separated on a 12% SDS−polyacrylamide gel and stained with Coomassie blue. Target bands were cut out, digested with trypsin, and subjected to mass spectrometric analysis following a previously described protocol [23].

### Statistical analysis

Statistically significant differences were identified by performing a one-way ANOVA followed by a two-tailed unpaired Student’s t test.

## Results

### αIIbβ3 and fibrinogen are required for platelet spreading, signal amplification, and thrombus formation over immobilized IgG

**Fig 1:**
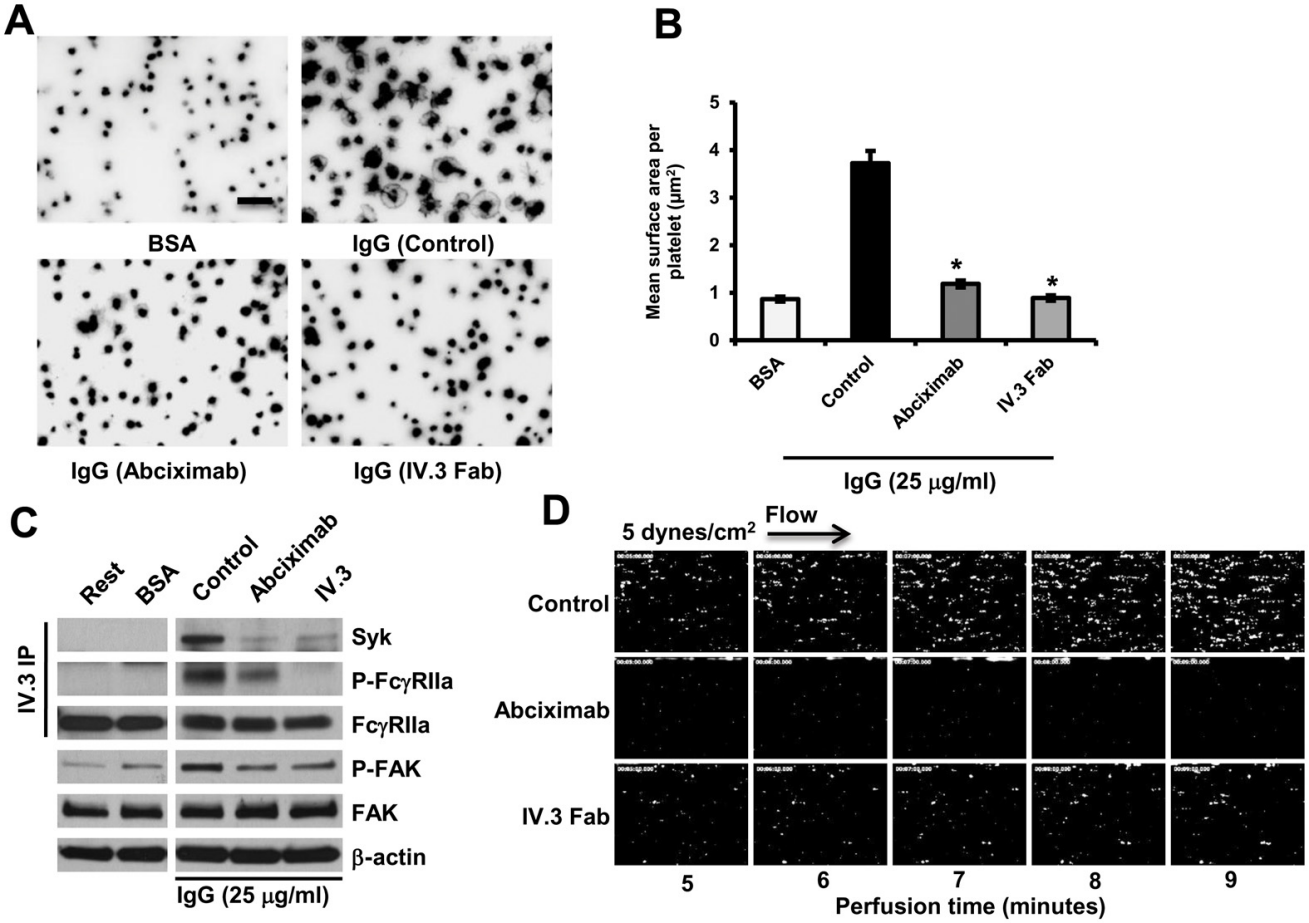
Blocking αIIbβ3-fibrinogen interactions prevents spreading of human platelets and thrombus formation over immobilized IgG. **(A)** Washed human platelets spread on BSA- or IgG-coated coverslips for 30 minutes in the presence or absence of the integrin αIIbβ3 antagonist abciximab (6.7 μg/ml) or Fab fragments of the FcγRIIa blocking antibody mAb IV.3 (10 μg/ml). Spread platelets were fixed, permeabilized and stained with rhodamine-phalloidin. Scale bar, 5μm. Images are representative of three independent experiments. **(B)** Platelet spreading was quantified using Metamorph software and shown as the mean μm^2^ ± SEM of at least 200 platelets/group from one of 3 representative experiments. (*P<0.05). Statistically significant differences were identified by performing a one-way ANOVA followed by a two-tailed Student’s t test. Note that abciximab or IV.3 Fab significantly inhibited platelet spreading on immobilized IgG. **(C)** mAb IV.3 immunoprecipitates of lysed spread platelets were analyzed by Western blot with the indicated antibodies. The blots for P-FAK and FAK were performed using whole cell lysates. Note that platelet binding to immobilized IgG elicits strong activation of FcγRIIa and Fak, as well as enhanced recruitment of Syk, and that both abciximab and mAb IV.3 inhibit platelet spreading-induced phosphorylation. **(D)** Mepacrine-labeled whole blood was perfused at a shear rate 5 dynes/cm^2^ over 100 μg/ml IgG-coated microchannels in the presence or absence of 10 μg/ml mAb IV.3 Fabs, 6.7 μg/ml abciximab, or an isotype-matched control Fab. Epifluorescence microscopic images of platelet adhesion and thrombus formation shown are representative of three separate experiments. Note that abciximab inhibits thrombus formation over immobilized IgG under conditions of flow.

We employed two complementary strategies to examine the potential contribution of αIIbβ3 in amplifying platelet responses downstream of their binding to immobilized IgG. In the first, human platelets were incubated in IgG-coated chambers under either static or flow conditions in the presence versus absence of the fibrinogen receptor antagonist, abciximab. As shown in Fig 1 abciximab blocked the spreading of human platelets on immobilized IgG nearly as well as did the FcγRIIa-specific mAb, IV.3 (Fig 1A and 1B). Small molecule antagonists of αIIbβ3-fibrinogen interactions like eptifibatide and tirofiban similarly blocked platelet spreading on immobilized IgG (S1A–S1B Fig). Tirofiban also markedly inhibited the spreading of FcγRIIa-positive transgenic *mouse* platelets on immobilized IgG (S2A–S2B Fig). Binding to immobilized IgG resulted in strong phosphorylation of FcγRIIa ITAM tyrosines, and concomitant recruitment or activation of the tyrosine kinase Syk in both human (Fig 1C) and mouse (S2C Fig) platelets. Both FcγRIIa ITAM phosphorylation and Syk recruitment were suppressed by abciximab (Fig 1C), consistent with the known amplification of platelet activation responses via ligand binding-induced outside-in signaling through αIIbβ3 [24,25]. Notably, pp125^Fak^, a reporter of integrin clustering downstream of αIIbβ3/fibrinogen interactions [26], also became phosphorylated (Fig 1C), suggesting that platelet/IgG interactions had stimulated secretion of fibrinogen from platelet α-granules, leading to ligand binding-dependent clustering of αIIbβ3 –a well-known inducer of FAK phosphorylation [26–29]. Consistent with the premise that αIIbβ3 requires fibrinogen to support cell spreading on immobilized IgG, CHO cells stably expressing both FcγRIIa and αIIbβ3 failed to spread on IgG-coated glass slides unless soluble fibrinogen was also present (S3 Fig). Finally, abciximab effectively blocked thrombus formation of whole blood, which contains ~3 mg/ml fibrinogen, that was passed over immobilized IgG-coated chamber slides under conditions of venous flow (Fig 1D)–conditions likely to be present when platelets encounter IgG immune complexes *in vivo*.

**Fig 2:**
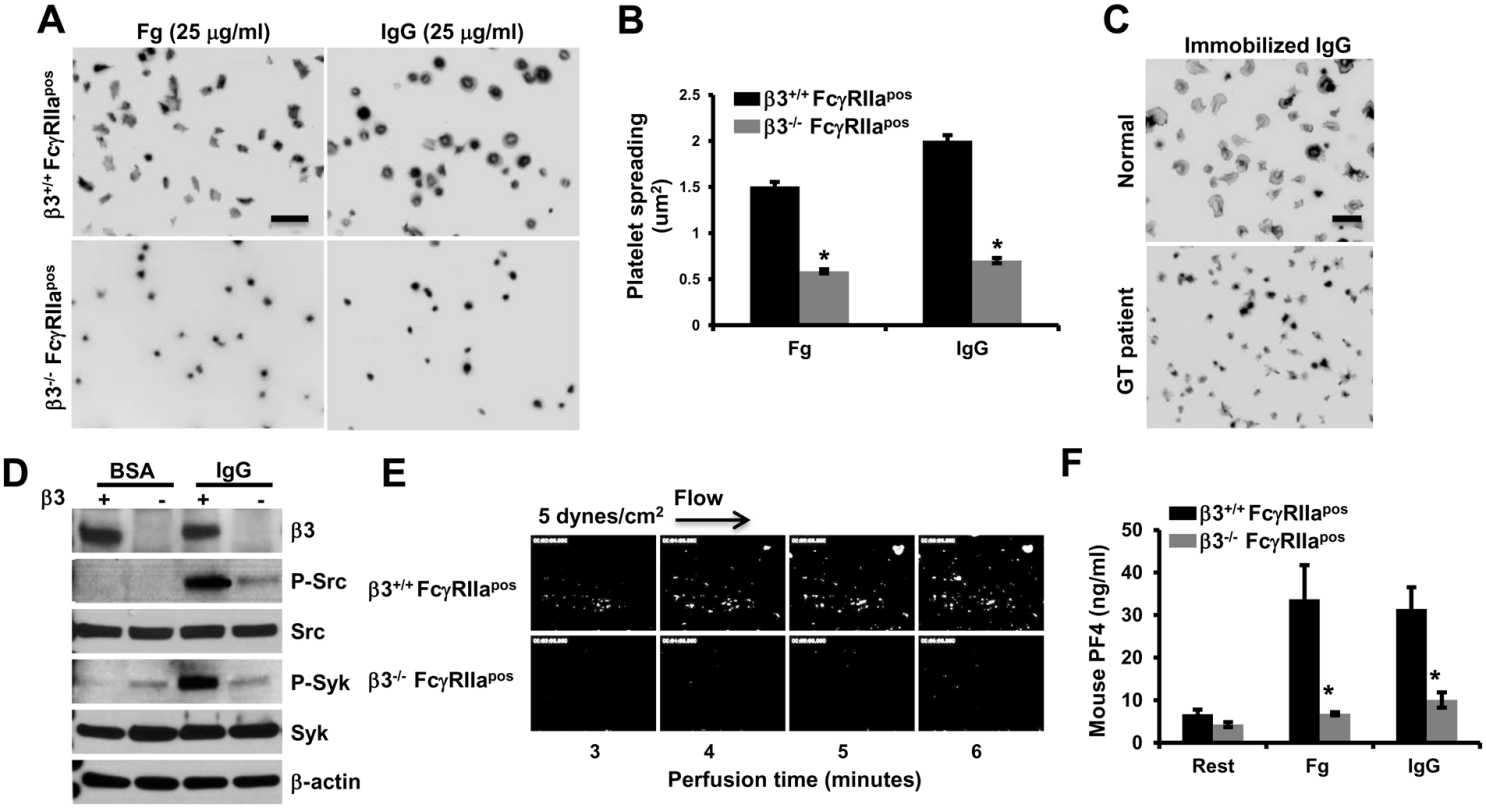
αIIbβ3-deficient platelets fail to spread, form thrombi, or efficiently secrete granule contents over immobilized IgG. **(A)** Washed platelets from αIIbβ3^-/-^ or αIIbβ3^+/+^ FcγRIIa^pos^ mice were allowed to spread and analyzed as described in the legend for Fig 1. Representative images of three independent experiments are shown. Scale bar, 5 μm. Note that αIIbβ3-deficient platelets failed to spread on fibrinogen, as expected, but also failed to spread on immobilized IgG. **(B)** Platelet spreading was quantified using Metamorph software and shown as the mean μm^2^ ± SEM of at least 200 platelets /group from one of 3 representative experiments (* *P*<0.01). Statistically significant differences were identified by performing a one-way ANOVA followed by a two-tailed Student’s t test. (**C**) Washed human platelets from a Type 1 Glanzmann thrombasthenic (GT) and a healthy volunteer were allowed to spread on IgG for 30 minutes at 37°C. Note that the control platelets formed filopodia and large lamellipodia, while GT platelets failed to spread on immobilized IgG. **(D)** Lysates of spread murine platelets were analyzed by Western blotting with the indicated antibodies. Note that αIIbβ3^-/-^/FcγRIIa^pos^ platelets show decreased activation of Src and Syk compared with αIIbβ3^+/+^/FcγRIIa^pos^ platelets. **(E)** Anticoagulated, mepacrine-labeled whole blood from αIIbβ3^+/+^/FcγRIIa^pos^ and αIIbβ3^-/-^/FcγRIIa^pos^ mice was perfused over 100 μg/mL IgG-coated flow chambers at a shear rate of 5 dynes/cm^2^ and images acquired using epifluorescence microscopy. Data shown are representative of three separate experiments. Note that αIIbβ3-deficient murine platelets exhibited dramatically-reduced thrombus formation compared to their wild-type counterparts. (**F**) PF4 secretion from washed murine platelets from αIIbβ3^-/-^/FcγRIIa^pos^ and αIIbβ3^+/+^/FcγRIIa^pos^ mice after 30 minute spreading BSA-, 25 μg/ml fibrinogen-, or 25 μg/mL IgG-coated glass slides. PF4 secreted into the culture supernatant was determined by ELISA. Note that secretion was markedly reduced in αIIbβ3^-/-^/FcγRIIa^pos^ platelets (* *P*<0.01). Statistically significant differences were identified by performing two-tailed Student’s t test.

To confirm the importance of αIIbβ3 in platelet reactions downstream of IgG-induced activation, the effects of αIIbβ3 deficiency on platelet reactivity to immobilized IgG were examined. As shown in Fig 2, both mouse (Fig 2A and 2B) and human (Fig 2C) αIIbβ3-deficient platelets spread poorly on immobilized IgG. Phosphorylation of Src and Syk induced by platelet spreading on immobilized IgG was also greatly diminished in platelets missing αIIbβ3 (Fig 2D), as was thrombus formation (Fig 2E) and α-granule secretion (Fig 2F).

### Involvement of Src- and Syk-family kinases in immobilized IgG-induced platelet activation

**Fig 3:**
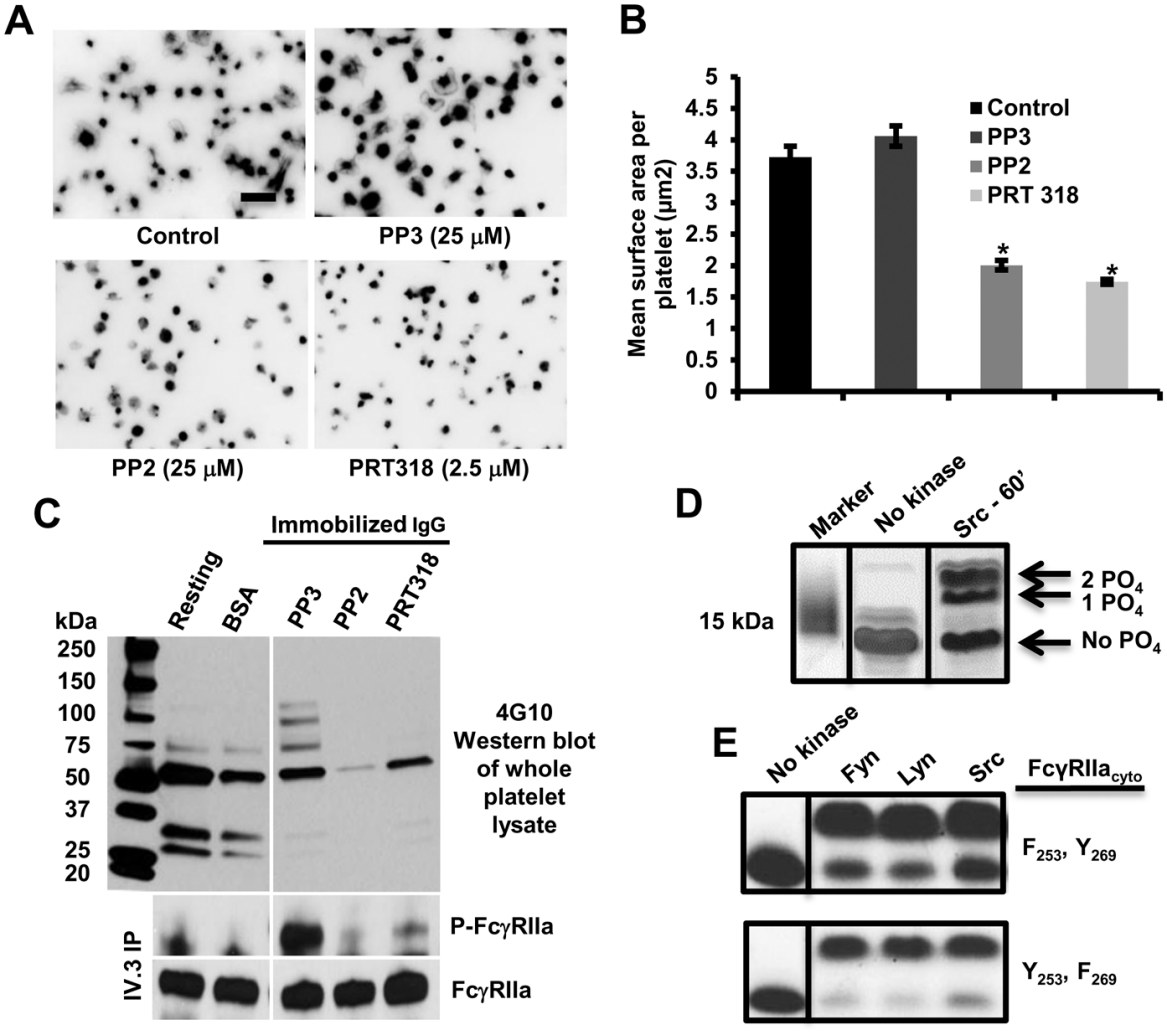
Role of Src- and Syk-family kinases in platelet activation by immobilized IgG. **(A)** Washed human platelets were incubated in IgG-coated microtiter chamber slides in the presence of DMSO (Control), PP3, PP2, or PRT318 for 30 minutes. Representative platelet spreading images of three independent experiments are shown. Scale bar, 5μm. **(B)** Quantitation shown is the mean μm^2^ ± SEM of at least 200 platelets /group from one of three representative experiments. Statistically significant differences were identified by performing a one-way ANOVA followed by a two-tailed Student’s t test. Note that both Src and Syk family kinases appear to be involved compared with DMSO vehicle- or PP3-treated platelets (*P <0.01). **(C)** Washed human platelets were incubated in IgG-coated plates in the presence of DMSO (Control), PP3, PP2, or PRT318 for 30 minutes. IV.3 immunoprecipitation and Western blot reveals that inhibitors of Src and Syk kinase had pronounced effects on early tyrosine phosphorylation events, including phosphorylation of FcγRIIa ITAM tyrosines. **(D)** Src-mediated phosphorylation of purified, recombinant FcγRIIa cytoplasmic domain (FcγRIIa_cyto_). Phosphorylated FcγRIIa_cyto_ was incubated for 60 minutes in the presence of purified Src + ATP. Coomassie blue staining of SDS-PAGE gels of the resulting products reveal both mono- and di-phosphorylated FcγRIIa_cyto_ species. Results are representative of two independent experiments. **(E)** Mutant forms of FcγRIIa_cyto_ containing only one of two ITAM tyrosines were incubated with Src, Fyn, or Lyn for 60 minutes, separated by SDS-PAGE and stained with Coomassie blue. Note that Src family kinases are able to phosphorylate either tyrosine residue independent of the phosphorylation state of the other ITAM tyrosine. Results are representative of two independent experiments.

The involvement of Src and Syk family kinases in cellular responses downstream from platelet-IgG interactions was examined using a series of kinase-specific inhibitors. As shown in Fig 3A and 3B, spreading of human platelets on IgG-coated microtiter wells was abolished in the presence of either the Src family kinase inhibitor, PP2, or the Syk kinase inhibitor PRT-060318 (PRT318) [30]. Spreading of FcγRIIa-positive transgenic mouse platelets on immobilized IgG was similarly affected by these two inhibitors (S4 Fig). In contrast, PP3, the inactive analogue of PP2, had no effect. PP2 and PRT318 also blocked tyrosine phosphorylation of multiple cellular tyrosine kinase substrates, including FcγRIIa itself (Fig 3C).

To determine whether Src-family kinases were capable of phosphorylating *both* ITAM tyrosine residues, a recombinant protein comprised of the entire FcγRIIa ITAM cytoplasmic domain (FcγRIIa_cyto_) was subjected to an *in vitro* kinase assay, its products separated by SDS-PAGE and then visualized by staining with Coomassie blue. As shown in Fig 3D, both mono- and di-phosphorylated FcγRIIa_cyto_ species were generated by Src. Kinase assays employing either Lyn or Fyn showed identical results (data not shown). To determine the identity of the ITAM tyrosine that became phosphorylated first, the lower bands from each of the three Src-family kinase reactions, thought to represent the mono-phosphorylated species, were cut out and subjected to trypsinization/mass spectrometry analysis. As shown in Table 1, peptides phosphorylated on either ITAM tyrosine residue—Y_253_ or Y_269_—were found to be derived from the lower MW band. That these two tyrosines are able to be phosphorylated independent of the phosphorylation state of the other was further shown by the ability of Fyn, Lyn, and Src to phosphorylate recombinant FcγRIIa cytoplasmic constructs in which either Y_253_ or Y_269_ had been mutated to phenylalanine (Fig 3E).

**Table 1:** Mass spectrometry analysis of FcγRIIa cytoplasmic domain phosphopeptides generated from *in vitro* kinase reactions.

Kinase used	Phosphopeptides detected
Src	QLEETNND**F**ETADGG**pY**_**253**_MT**L**NPR
	APTDDDKNI**pY**_**269**_LT**L**PPNDHVNSNN
	NI**pY**_**269**_LT**L**PPNDHVNSNN
Lyn	QLEETNND**F**ETADGG**pY**_**253**_MT**L**NPR
	APTDDDKNI**pY**_**269**_LT**L**PPNDHVNSNN
	NI**pY**_**269**_LT**L**PPNDHVNSNN
Fyn	QLEETNND**F**ETADGG**pY**_**253**_MT**L**NPR
	APTDDDKNI**pY**_**269**_LT**L**PPNDHVNSNN
	NI**pY**_**269**_LT**L**PPNDHVNSNN

Recombinant full-length FcγRIIa cytoplasmic domain: CRKKRISANSTDPVKAAQFEPPGRQQMIAIRK*R*QLEETNNDYETADGG**YMTL**NP*R*APTDDD*K*NI**YLTL**PPNDHVNSNN was subjected to an *in vitro* kinase reaction, digested with trypsin, and subjected to mass spec analysis. The above phosphopeptides were detected. Note that both ITAM tyrosines 253 and 269 are targets for SFKs, at least using peptides as a substrate. The YxxL ITAM motifs are delimited with red bold letters, while the tryptic cleavage sites are underlined and italicized. The naturally-occurring non-ITAM tyrosine at residue 246 was mutated in the recombinant protein to a phenylalanine to prevent its phosphorylation.

**Fig 4:**
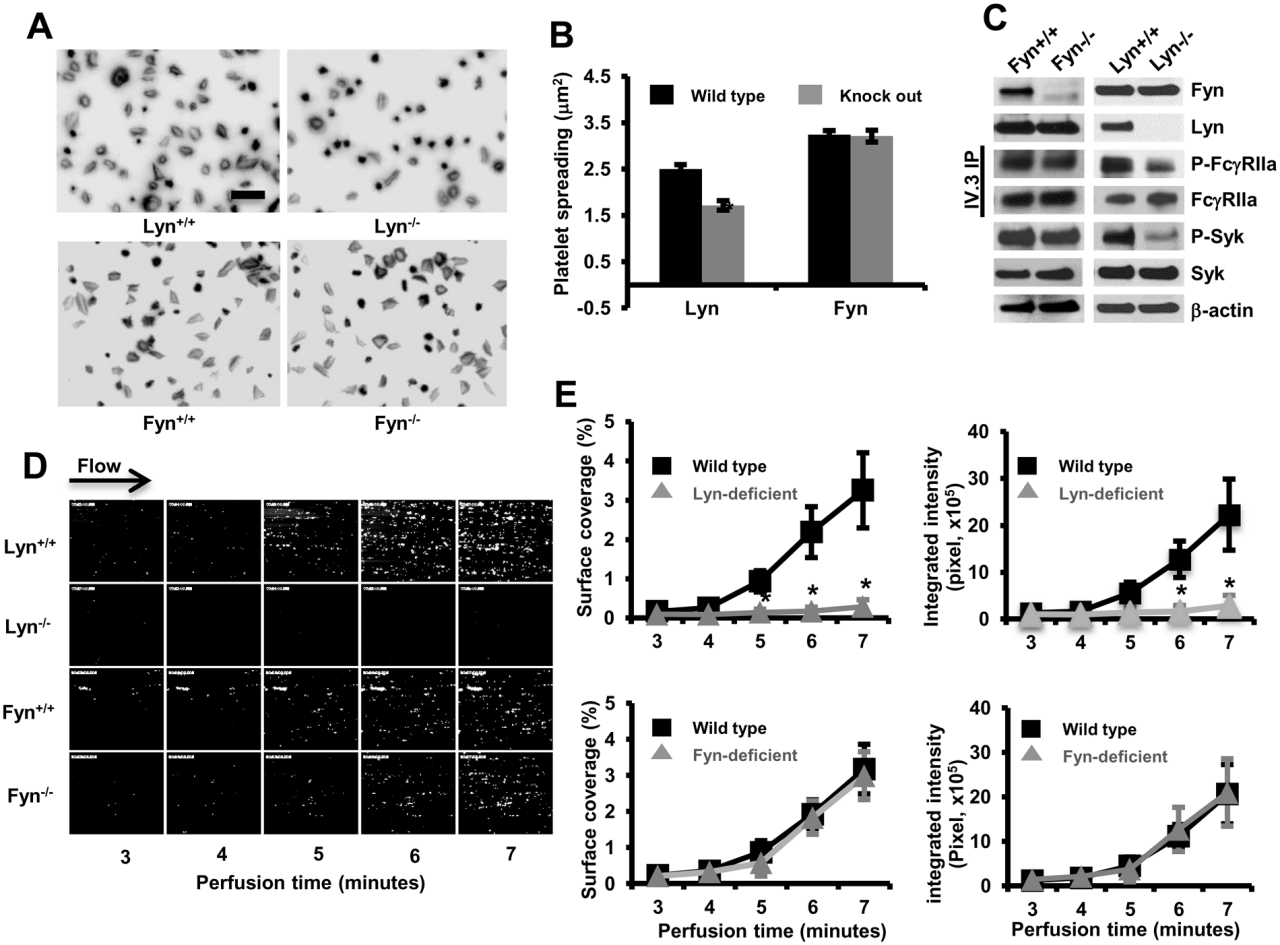
Lyn, but not Fyn, is required for integrin-dependent platelet spreading and thrombus formation over immobilized IgG. **(A)** Washed FcγRIIa transgenic mouse platelets lacking the Src-family kinase Lyn or Fyn were plated on immobilized IgG-coated coverslips. After 30 minutes, spread platelets were fixed, permeabilized and stained. Images are representative of three independent experiments. **(B)** Quantification of platelet surface area of at least 200 platelets/group using Metamorph software as described above (* *P*<0.05). Results are reported as mean ± S.E.M from one of three representative experiments. Fyn- and Lyn-deficient mice are on different strains (see Methods) and thus exhibit different degrees of spreading, even on wild-type backgrounds. Note that only Lyn-deficient platelets exhibit a spreading defect. **(C)** mAb IV.3 immunoprecipitates of lysed spread platelets were analyzed by Western blot with the indicated antibodies. The blots for (Fyn, Lyn, p-Syk, Syk, β-actin) were performed using whole cell lysates. Results were representative of two independent experiments. Note that tyrosine phosphorylation of FcγRIIa and Syk is reduced in Lyn^-/-^, but not Fyn^-/-^, FcγRIIa^pos^ platelets during spreading on immobilized IgG. **(D)** Whole blood from FcγRIIa transgenic mice lacking Lyn or Fyn was perfused at 5 dynes/cm^2^ over IgG-coated coverslips and images acquired using epifluorescence microscopy. **(E)** Quantification of platelet thrombi expressed as the percentage of total area covered by thrombi (left panels) or total integrated fluorescence intensity (right panels) was performed using Metamorph program. Statistical analysis was performed using the Student’s t test, and data represented as mean ± S.E.M (n = 3 per group). Note that thrombus formation was significantly inhibited (*P<0.*05)* in FcγRIIa^pos^ platelets lacking Lyn (but not Fyn).

To determine the tyrosine kinase responsible for activation of intact platelets downstream of encountering immobilized IgG, we crossed FcγRIIa^pos^ mice with Lyn- or Fyn-deficient mice. The expression levels of FcγRIIa were comparable among different groups (flow-cytometry data not shown). We compared the ability of platelets to spread and form thrombi over immobilized IgG. As shown in Fig 4A and 4B, whereas Fyn^-/-^/FcγRIIa^pos^ platelets spread normally, spreading of Lyn^-/-^/FcγRIIa^pos^ platelets was markedly impaired, despite normal expression of Fyn (Fig 4C) and Src (not shown). Tyrosine phosphorylation of FcγRIIa and Syk in Fyn^-/-^/ FcγRIIa^pos^ platelets was also comparable to that observed in Fyn^+/+^/FcγRIIa^pos^ platelets; however, compared with Lyn^+/+^/FcγRIIa^pos^ platelets, platelets from Lyn^-/-^/FcγRIIa^pos^ exhibited significantly reduced tyrosine phosphorylation of FcγRIIa and Syk, again despite normal expression of Fyn and Src. Finally, when whole blood from Fyn^+/+^/FcγRIIa^pos^, Fyn^-/-^/FcγRIIa^pos^, Lyn^-/-^/FcγRIIa^pos^, or Lyn^-/-^/FcγRIIa^pos^ was subjected to microfluidic flow conditions, only Lyn^-/-^/FcγRIIa^pos^ blood failed to form thrombi (Fig 4D and 4E).

## Discussion

IgG immune complexes contribute to the etiology and pathogenesis of numerous autoimmune disorders, including heparin-induced thrombocytopenia, systemic lupus erythematosus, rheumatoid- and collagen-induced arthritis, and chronic glomerulonephritis. Patients suffering from immune complex-related disorders are known to be susceptible to platelet-mediated thrombotic events. Though platelet activation and signal transduction pathways initiated by the binding of IgG immune complexes to its platelet receptor, FcγRIIa, are well understood, the role of the major platelet adhesion receptor, integrin αIIbβ3, in amplifying platelet activation and mediating adhesion and aggregation downstream of encountering IgG immune complexes is not known.

The purpose of the present investigation was to gain further insight into the molecular requirements for activation, spreading and thrombus formation in platelets encountering IgG immune complexes. We used small molecule antagonists of αIIbβ3, as well as human and mouse platelets that selectively express αIIbβ3 and FcγRIIa, to determine whether the integrin αIIbβ3 is required for platelet spreading and thrombus formation over immobilized IgG. We found that while FcγRIIa-IgG interactions are sufficient to support initial adhesion and stimulate limited platelet activation and secretion, events downstream of encountering immobilized IgG, including amplification of the release reaction, cell spreading and thrombus formation, require fibrinogen binding to its αIIbβ3 receptor.

Previous studies have shown crosslinking FcγRIIa leads to activation of the Src-family kinases Src, Lyn, and to a lesser extent Fyn [31], as well as the protein tyrosine kinase Syk [15]. The involvement of these families of tyrosine kinases in FcγRIIa signaling is strikingly similar to the molecular requirements for platelets spreading on immobilized fibrinogen in that both Src and Syk family kinases have been known for nearly 20 years to play a significant role in outside-in signaling and platelet spreading [32–34]. Two members of the Src family, Src and Fyn, are directly associated with distinct regions of the integrin β3 cytoplasmic domain [35,36], and clustering of αIIbβ3 has been shown to induce direct activation of Src [37]. Notably, while deficiency of Src leads to profound defects in αIIbβ3-mediated platelet spreading on immobilized fibrinogen, absence of Lyn actually promotes platelet spreading [38,39]. The roles of Src, Fyn, and Lyn on platelet spreading on immobilized IgG, on the other hand, remain to be defined.

Dual ITAM and ITIM-containing proteins have the capacity to become processively phosphorylated—i.e. the first tyrosine residue, once phosphorylated, promotes high-affinity recruitment of the same, or a different, SH2 domain-containing kinase that then goes on to carry out efficient phosphorylation of the second tyrosine [40]. We have recently described such a mechanism for phosphorylation of the two ITIM tyrosines of the inhibitory receptor, PECAM-1, in which phosphorylation of Y_686_ by the Src-family kinase, Lyn, is a necessary prerequisite for recruitment of Csk (C-terminal Src kinase) and its subsequent phosphorylation of Y_663_ [23,41,42]. Both Lyn and Syk have been shown in *in vitro* kinase assays to be capable of tyrosine phosphorylating a recombinant FcγRIIa cytoplasmic domain [43], and therefore, represent the most likely candidate FcγRIIa ITAM kinases. Whether they exhibit similar sequence specificity and/or are reliant on sequential phosphorylation, however, has not been previously described. We found that inhibitors of Src and Syk kinase had pronounced effects on phosphorylation of FcγRIIa ITAM tyrosines, however, unlike the ITIMs of PECAM-1, the Src-family kinases involved in FcγRIIa tyrosine phosphorylation exhibit no sequence specificity, and FcγRIIa ITAM tyrosines are independently, rather than sequentially, phosphorylated, at least *in vitro* (Fig 3). Moreover, Lyn, but not Fyn or Src, appears to be required for initial platelet activation over immobilized IgG. These findings are in stark contrast to the kinase requirements for platelet spreading on immobilized fibrinogen, which requires Src, but not Lyn [38,39].

Since Lyn has an inhibitory role in αIIbβ3-mediated platelet spreading on immobilized fibrinogen [38,39], what might explain its essential role in supporting platelet spreading and thrombus formation on immobilized IgG? The answer, at least in part, may lie in the recent observation of Li et al., who found that Lyn is required for α-granule secretion [44]. Taken together with (1) the finding that fibrinogen is a necessary substrate for cells to spread on IgG-coated surfaces (reference [45], Fig 1, and S3 Fig), (2) the ability of Lyn to carry out robust phosphorylation of both FcγRIIa ITAM tyrosines (Fig 3), and (3) the diminished recruitment of Syk to FcγRIIa ITAMs in Lyn^-/-^ platelets (Fig 4C), it seems likely that Lyn is the physiologic FcγRIIa ITAM kinase responsible for initial platelet secretion and integrin activation following platelet/IgG interactions.

Platelet adhesion and aggregation at sites of vascular injury are essential for hemostasis, and ligand binding to integrins and immunoreceptor family members trigger signaling pathways that increasingly appear to share common components that converge to accomplish a common goal, namely that of integrin activation, granule release and controlled thrombus formation. In the present study, we have further defined the molecular requirements for platelet activation following their encounter with immune complexes. When cell surface FcγRIIa binds the Fc region of immobilized IgG, Lyn kinases located near FcγRIIa [46], perhaps due to their co-enrichment in lipid rafts [39,47] become activated and phosphorylate the ITAM tyrosines of FcγRIIa, initiating activation of PLCγ2 and generation of second messengers that result in Ca^++^ mobilization, secretion of α-granule fibrinogen, and activation of αIIbβ3. Under the artificial *in vitro* conditions used for platelet spreading assays, which take place in the absence of extracellular fibrinogen, secreted α-granule-derived fibrinogen becomes the substrate that supports αIIbβ3-mediated platelet spreading (Fig 1A and 1B, Fig 2A and 2C). Blocking αIIbβ3 with antagonists of αIIbβ3/Fg interactions (Fig 1) or deficiency of αIIbβ3 (Fig 2) totally abolishes the ability of platelets to spread on immobilized IgG. In the case of whole-blood, αIIbβ3, activated as a result of FcγRIIa/immune complex interactions, is absolutely required for thrombus formation (Figs 1D and 2E). These novel and somewhat unexpected observations extend previous notions about functionally-important integrin/ITAM connections, and provide compelling rationale for future clinical studies to determine whether anti-αIIbβ3-directed therapeutics might benefit patients suffering from immune complex disorders in which thrombosis may be a complicating condition. In addition, the demonstrated requirement for specific tyrosine kinases in these events (Figs 3 and 4) suggests that Syk inhibitors already currently in clinical trials [48,49] may have the added benefit of suppressing not only the immune response responsible for immune complex formation, but also the confounding platelet activation events that occur downstream of platelet/immune complex interactions.

## Supporting Information

S1 Fig Small molecule antagonists of aIIbb3-fibrinogen interactions inhibit spreading of human platelets on immobilized IgG. **(A)** Washed platelets from human blood were incubated with BSA- or IgG-coated coverslips for 45 minutes in the presence or absence of the integrin αIIbβ3 antagonists Eptifibatide (6.7 mg/ml) or Tirofiban (10 mg/ml). After spreading, platelets were fixed, permeabilized and stained with rhodamine-phalloidin. Images are representative of three independent experiments. Scale bar, 5μm. (B) Platelet spreading was quantified using Metamorph software and shown as the mean μm^2^ ± SEM of at least 200 platelets/group from one of 3 representative experiments. (*P<0.01). Statistically significant differences were identified by performing a two-tailed Student’s t test. Note that Eptifibatide or Tirofiban significantly inhibited platelet spreading on immobilized IgG.
(PDF)

S2 Fig Small molecule antagonists of αIIbβ3-fibrinogen interactions inhibit spreading of FcγRIIa^pos^ transgenic mouse platelets on immobilized IgG. **(A)** Washed platelets from FcγRIIa^pos^ mice were incubated over IgG-coated coverslips in the presence or absence of the integrin αIIbβ3 antagonist Tirofiban (10 μg/ml) for 30 minutes at 37°C. Platelets were then fixed, permeabilized and stained with rhodamine-phalloidin. Negative controls included spreading on BSA, or spreading in the presence of mAb IV.3 Fab fragments, which are known to block IgG/FcγRIIa interactions. Images are representative of three independent experiments. Scale bar, 5μm. (B) Platelet spreading was quantified using Metamorph software and shown as the mean μm^2^ ± SEM of at least 200 platelets/group from one of 3 representative experiments. (*P<0.01). Statistically significant differences were identified by performing a two-tailed Student’s t test. Note that Tirofiban significantly inhibited platelet spreading on immobilized IgG. **(C)** Lysates of platelets prepared as in panel A was subjected to mAb IV.3 immunoprecipitation/Western blot analysis using the indicated antibodies. Note that platelets show strong activation of FcγRIIa and Syk after binding to immobilized IgG, and that Tirofiban inhibits spreading-induced phosphorylation of both proteins. Results are representative of two independent experiments.
(PDF)

S3 Fig FcγRIIa binding to immobilized IgG is insufficient to support cell spreading. Chinese Hamster Ovary (CHO) cells stably expressing both αIIbβ3 and FcγRIIa were incubated with glass slides that had been coated with 25 μg/ml fibrinogen, 25 μg/ml IgG, or 25 μg/ml of IgG to which 250 μg/ml of soluble fibrinogen was added at the time of the assay. Images of cell spreading shown are representative of three independent experiments. Note that cell spreading is dependent upon αIIbβ3 binding to either immobilized or co-added fibrinogen for spreading to occur.
(PDF)

S4 Fig Src- and Syk-family kinase inhibitors block spreading of FcγRIIa^pos^ mouse platelets on immobilized IgG. **(A)** Washed FcγRIIa^pos^ platelets were added to IgG-coated microtiter chamber slides in the presence of the indicated reagents, and allowed to adhere and spread for 30 minutes at 37°C. Representative platelet spreading images of three independent experiments are shown. Scale bar, 5μm. Platelet spreading was quantified **(panel B)** using Metamorph software, with each bar representing the mean μm^2^ ± SEM of at least 200 platelets/group from one of 3 representative experiments. Statistically significant differences were identified by performing a one-way ANOVA followed by a two-tailed Student’s t test. (*P < 0.01, compared with DMSO-treated control platelets.**)** Note that preincubation of murine platelets with SFK and Syk inhibitors significantly inhibited platelet spreading on immobilized IgG.
(PDF)
