# Supplementary figures and images for: Platelet Activation and Thrombus Formation over IgG Immune Complexes Requires Integrin αIIbβ3 and Lyn Kinase
Source: PLoS One. 2015 Aug 20;10(8):e0135738. doi: 10.1371/journal.pone.0135738 (PMC4546160; doi:10.1371/journal.pone.0135738)

## Supplemental Figure 1

**A**

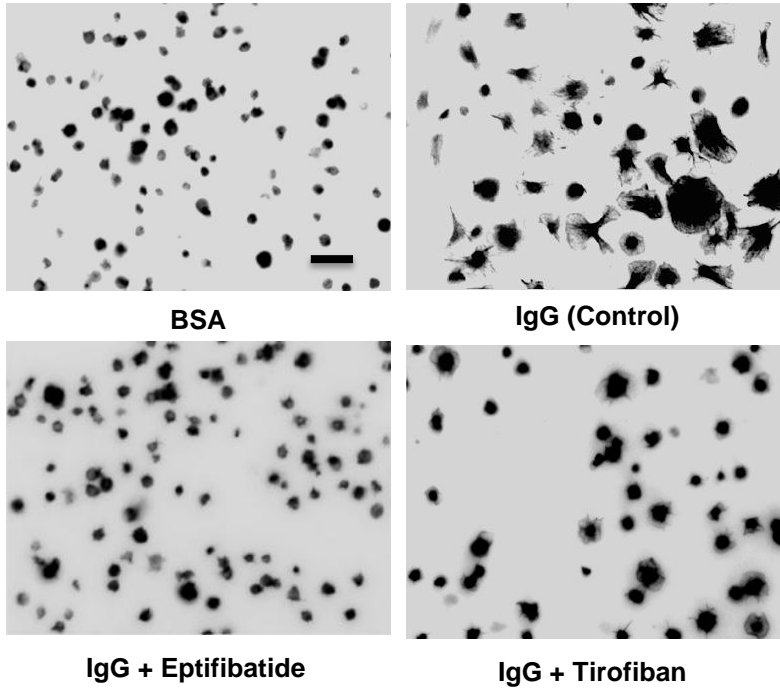

**B**

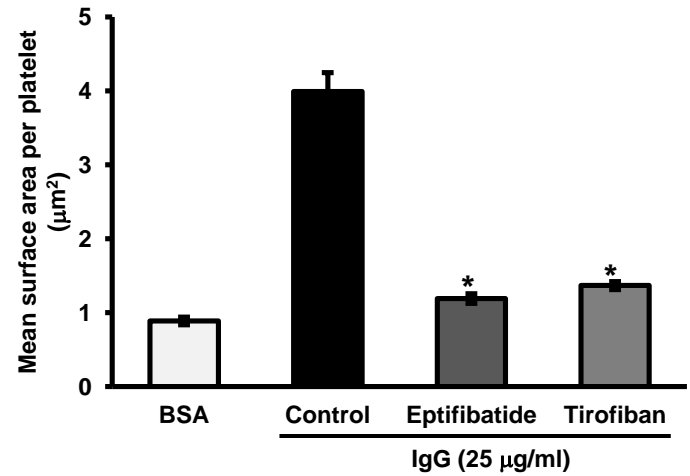

Supplement: S1 Fig — (A) Washed platelets from human blood were incubated with BSA- or IgG-coated coverslips for 45 minutes in the presence or absence of the integrin αIIbβ3 antagonists Eptifibatide (6.7 mg/ml) or Tirofiban (10 mg/ml). After spreading, platelets were fixed, permeabilized and stained with rhodamine-phalloidin. Images are representative of three independent experiments. Scale bar, 5μm. (B) Platelet spreading was quantified using Metamorph software and shown as the mean μm2 ± SEM of at least 200 platelets/group from one of 3 representative experiments. (*P<0.01). Statistically significant differences were identified by performing a two-tailed Student’s t test. Note that Eptifibatide or Tirofiban significantly inhibited platelet spreading on immobilized IgG. (PDF) [file pone.0135738.s001.pdf]

## Supplemental Figure 2

**A**

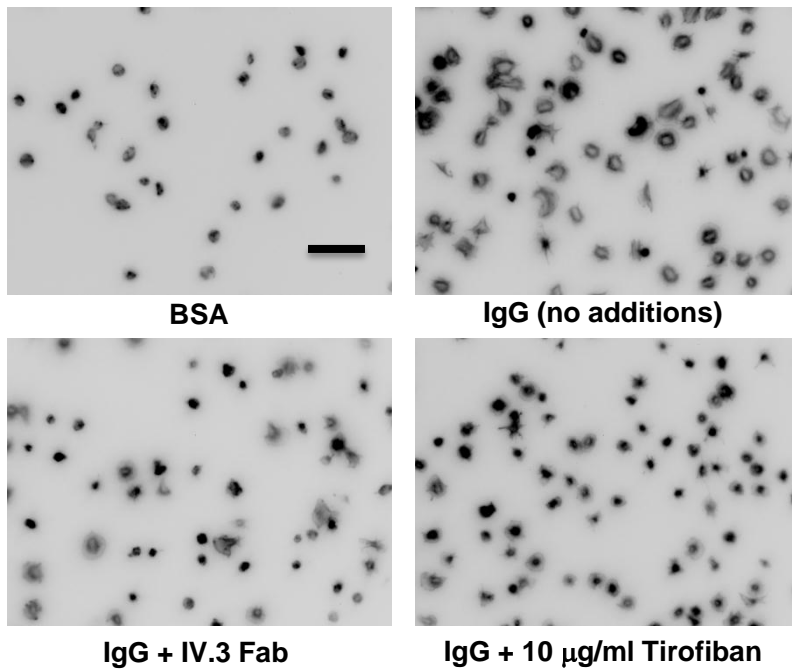

**B**

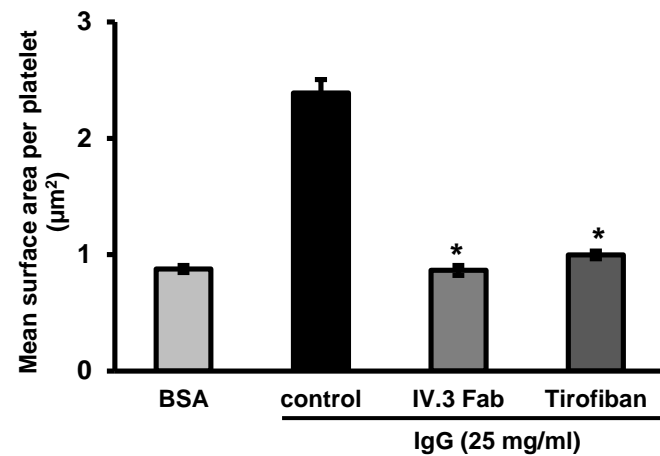

**C**

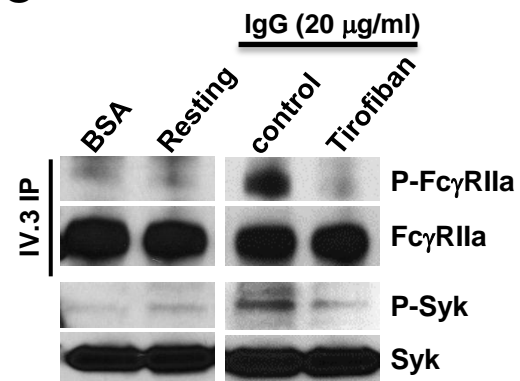

Supplement: S2 Fig — (A) Washed platelets from FcγRIIapos mice were incubated over IgG-coated coverslips in the presence or absence of the integrin αIIbβ3 antagonist Tirofiban (10 μg/ml) for 30 minutes at 37°C. Platelets were then fixed, permeabilized and stained with rhodamine-phalloidin. Negative controls included spreading on BSA, or spreading in the presence of mAb IV.3 Fab fragments, which are known to block IgG/FcγRIIa interactions. Images are representative of three independent experiments. Scale bar, 5μm. (B) Platelet spreading was quantified using Metamorph software and shown as the mean μm2 ± SEM of at least 200 platelets/group from one of 3 representative experiments. (*P<0.01). Statistically significant differences were identified by performing a two-tailed Student’s t test. Note that Tirofiban significantly inhibited platelet spreading on immobilized IgG. (C) Lysates of platelets prepared as in panel A was subjected to mAb IV.3 immunoprecipitation/Western blot analysis using the indicated antibodies. Note that platelets show strong activation of FcγRIIa and Syk after binding to immobilized IgG, and that Tirofiban inhibits spreading-induced phosphorylation of both proteins. Results are representative of two independent experiments. (PDF) [file pone.0135738.s002.pdf]

## Supplemental Figure 3

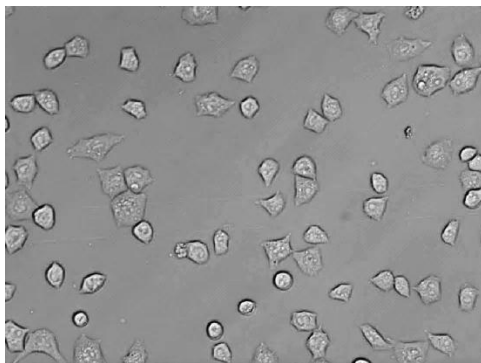

**Fg alone**

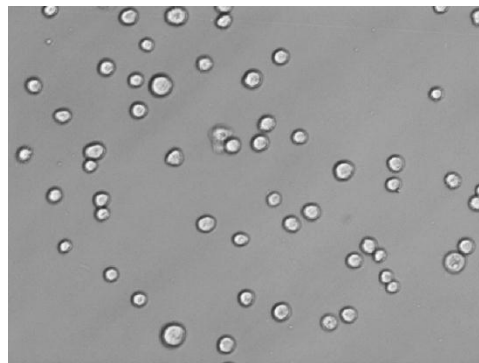

**IgG alone**

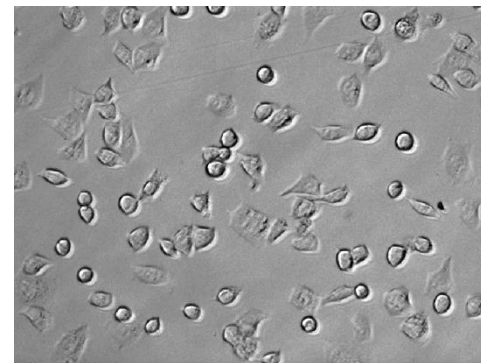

**IgG + soluble Fg**

Supplement: S3 Fig — Chinese Hamster Ovary (CHO) cells stably expressing both αIIbβ3 and FcγRIIa were incubated with glass slides that had been coated with 25 μg/ml fibrinogen, 25 μg/ml IgG, or 25 μg/ml of IgG to which 250 μg/ml of soluble fibrinogen was added at the time of the assay. Images of cell spreading shown are representative of three independent experiments. Note that cell spreading is dependent upon αIIbβ3 binding to either immobilized or co-added fibrinogen for spreading to occur. (PDF) [file pone.0135738.s003.pdf]

## Supplemental Figure 4

**A**

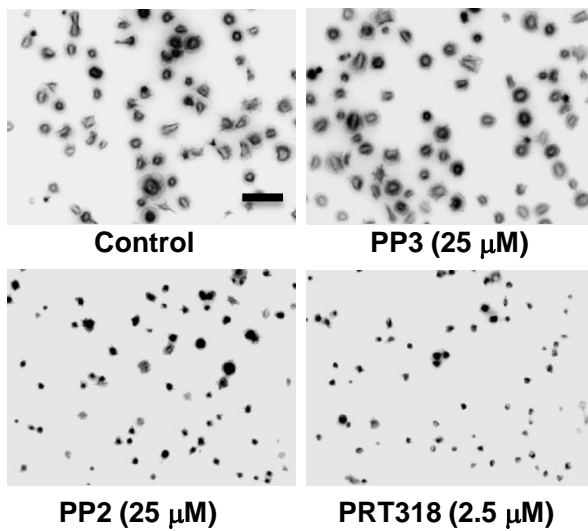

**B**

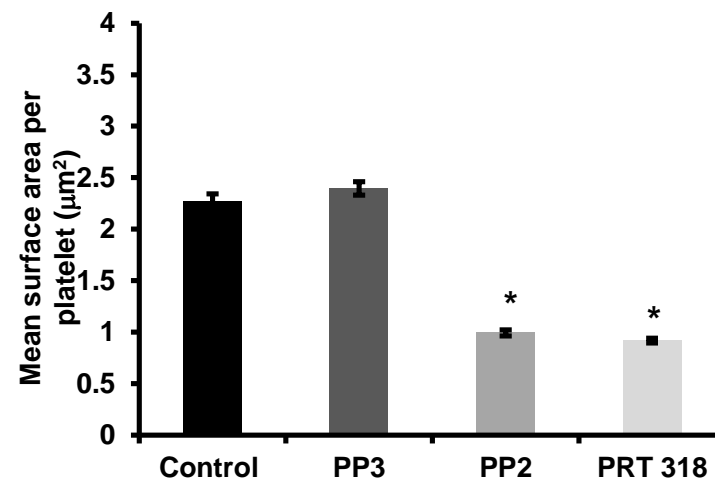

Supplement: S4 Fig — (A) Washed FcγRIIapos platelets were added to IgG-coated microtiter chamber slides in the presence of the indicated reagents, and allowed to adhere and spread for 30 minutes at 37°C. Representative platelet spreading images of three independent experiments are shown. Scale bar, 5μm. Platelet spreading was quantified (panel B) using Metamorph software, with each bar representing the mean μm2 ± SEM of at least 200 platelets/group from one of 3 representative experiments. Statistically significant differences were identified by performing a one-way ANOVA followed by a two-tailed Student’s t test. (*P < 0.01, compared with DMSO-treated control platelets.) Note that preincubation of murine platelets with SFK and Syk inhibitors significantly inhibited platelet spreading on immobilized IgG. (PDF) [file pone.0135738.s004.pdf]
